# What Elevation Makes 2.5D Prints Perceptually Natural?

**DOI:** 10.3390/ma15103573

**Published:** 2022-05-17

**Authors:** Altynay Kadyrova, Marius Pedersen, Stephen Westland

**Affiliations:** 1 Department of Computer Science, Norwegian University of Science and Technology, 2802 Gjøvik, Norway; marius.pedersen@ntnu.no; 2 School of Design, University of Leeds, Leeds LS2 9JT, UK; s.westland@leeds.ac.uk

**Keywords:** naturalness, 2.5D printing, elevation, wood images

## Abstract

Elevation plays a considerable role in naturalness perception of 2.5D prints. The necessary level of elevation to make 2.5D prints look perceptually natural may vary from application to application. Therefore, one needs to know the right elevation for specific applications to make the prints look perceptually natural. In this work, we investigated what elevation makes 2.5D prints of wood images perceptually natural. We worked with various wood content images such as wooden wicker, wall, roof, and floor. We found that the optimal elevation that makes 2.5D prints of wood images perceptually natural is content-dependent and in a range between 0.3 mm and 0.5 mm. Moreover, we found that the optimal elevation becomes 0.5 mm if we consider images of wood regardless of the wood content. In addition, there was a high correlation between majority of observers on naturalness perception of 2.5D prints of wood images.

## 1. Introduction

Naturalness of 2.5D prints is important for industry because 2.5D prints need to provide realistic representation of the content they depict. Naturalness perception of 2.5D prints can be affected by various factors (e.g., illumination, viewing angle, user experience, ink types, etc.) and the quality attributes of the prints (e.g., elevation, color, gloss, etc.). For example, Kadyrova et al. [1] found that elevation affects the naturalness perception of 2.5D prints.

Elevation level tends to change perceived appearance aspects such as the naturalness of 2.5D prints. We found from our previous work that observers perceive 2.5D prints of wood images to be natural at lower elevation levels [1]. As a result, it is relevant to investigate the exact elevation level that is perceived as natural for 2.5D prints of wood images. Hence, this work seeks to provide detailed information on this. We focus on various elevation levels at a given viewing distance and illumination. Images of wood material were selected because wood is a familiar material for most people and has a variety of forms [2]. Our work may provide industry with valuable insights on what elevation levels to use for 2.5D prints of wood images’ content so that they are perceived as natural. Wood is also a commonly used reproduced material in decor printing and is therefore also industry relevant. It is important to mention that, in the case of 2.5D prints, elevation can be synonymous with height, relief, depth, and similar attributes and expressions [1,3]. Thus, we do not differentiate definitions of, for example, elevation and height as in geography or other fields. By elevation we mean a height that is a distance from the bottom (i.e., substrate) to the top (i.e., raised point).

This paper is organized as follows: background information about the naturalness perception of 2.5D prints is given first; methodology is given afterwards followed by the results and discussion; last, conclusions and suggestions for future work are given.

## 2. Background

Naturalness perception tends to be multidimensional [2]. In other words, it is complex and varies depending on what is examined as natural. For example, considerable research has been carried out to explore wood’s naturalness perception [2,4,5] and the impact of wood on human well-being [6,7,8]. Overvliet and Soto-Faraco [2] studied the impact of vision and touch, and their combination on naturalness perception of wood samples with varying treatment levels through four psychophysical methods (ranked ordering, binary decision, magnitude estimation, and labelled scaling). They found consistent results across the four methods. They also found that visual and tactile assessments show a high correlation with visuo-tactile assessment. However, they mentioned that it is challenging to separate the effects of vision and tactile assessment on material’s naturalness perception. Strobel et al. [9] investigated the link between wood’s physical properties and general knowledge on interior wood products. They found several physical properties that their observers used for wood assessment (e.g., grain, color, chemical composition, etc.) as well as other properties that had impact on wood use in interior such as noise, scent, flammability, warmth, and feeling. They further stated that scent and grain are the two main properties that impact on naturalness perception of wood. Their results are important for the reproduction of wooden materials. In particular, feeling wood is the key property for the reproduction of wooden materials that should be considered by industry.

Some wood types can be expensive and therefore their realistic reproduction can be helpful in different applications, especially in decor or in education for architecture students. The reproduction can be achieved in different ways. For decor, the relevant technique for reproduction can be 2.5D printing. It allows superimposition of successive ink layers to create surface relief (i.e., elevation) and can achieve better fine detail reproduction than 3D printing [10]. In other words, 2.5D printing creates slightly elevated prints. To reproduce wood via 2.5D printing, one needs to input wood images and their height maps, and desired elevation level (additionally, one can define settings for gloss and color parameters). The output is 2.5D prints of wood images fabricated at the desired elevation level. The substrate for printing can be real wood or other substitutes depending on the need. One can use images of any material (e.g., stone, metal) to reproduce those materials via 2.5D printing. It is reasonable to assess naturalness perception of the 2.5D reproductions/prints of different material images because one might be curious if 2.5D printing was able to realistically reproduce, for example, wood images on either wood or another substrate.

There is limited research that has investigated the naturalness perception of 2.5D prints. The work of Kadyrova et al. [1] demonstrates the effect of elevation and surface roughness on the naturalness perception of 2.5D decor prints. The elevation (i.e., height) levels they used were 0.4 mm, 0.6 mm, and 0.8 mm while the surface roughness was considered as a height difference within a local neighborhood. They found that there is an impact by elevation rather than surface roughness on the naturalness perception of 2.5D decor prints. Moreover, they found that the naturalness of 2.5D decor prints is content-dependent with regard to the elevation effect. Images of four material categories were examined in their work. They were wood, glass, stone, and metal, and 2.5D prints were fabricated on an outdoor paper substrate. Based on their results, the observers found lower elevation as natural for wood and glass 2.5D prints. For the stone and metal categories, they did not find a clear tendency. They also provided perceptual attributes that one tends to use during naturalness assessment of 2.5D prints. They defined three levels of perceptual attributes. There were twelve main attributes in total. Color, roughness, gloss, elevation, and lightness were the top five. Furthermore, they found that the most used perceptual attributes among the four examined material categories were color, roughness, and gloss.

The quality attributes most commonly used for the quality assessment of 2.5D prints were studied by Kadyrova et al. [3]. The top five were color, sharpness, elevation, lightness, and naturalness. This shows that observers tend to look at elevation and naturalness aspects of 2.5D prints during quality assessment. Hence, this supports the point that it is important to investigate the naturalness perception of 2.5D prints with respect to the elevation. Furthermore, they observed the following factors that might affect the quality assessment of 2.5D prints: content, aesthetic appearance, and previous knowledge and experience.

**Figure 1:**
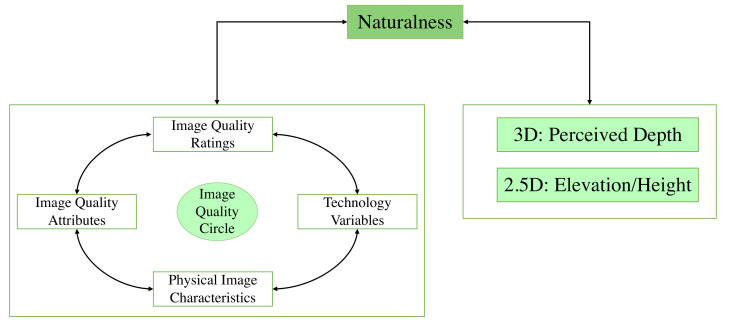
Slight modification proposal of Heynderickx [11] model with regard to 2.5D prints. We propose to consider elevation (or height) as the representative attribute that affects the naturalness perception of 2.5D prints in this model.

A model for assessing 3D display visual performance proposed by Heynderickx [11] shows that naturalness spans both perceived depth and image quality. In the case of 2.5D prints, observers tend to use depth to mean elevation [1,3]. Hence, we can view depth as elevation in this model for 2.5D prints’ case or we can slightly modify the model for 2.5D prints’ case as illustrated in Figure 1. The elevation is the main feature of 2.5D prints and it impacts the naturalness perception of 2.5D prints [1]. As a result, it can be considered as the representative attribute that describes the naturalness perception of 2.5D prints.

To conclude, the literature supports the need to explore the effect of elevation on the naturalness perception of 2.5D prints.

## 3. Methodology

**Figure 2:**
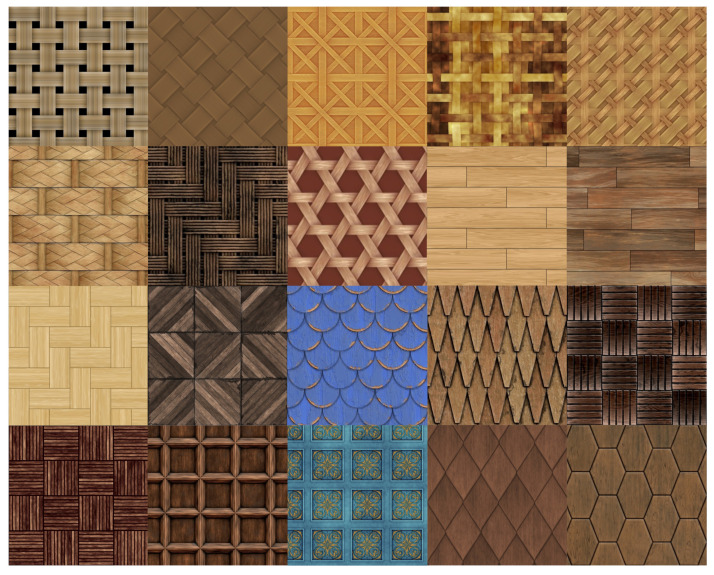
Wood images used in our work. From top left: 8 wicker, 4 floor, 2 roof, and 6 wall images.

Following our previous work [1], we refer to the term realistic representation of a print for the definition of naturalness. We considered 20 wood images with various wood content. We included images of wooden wicker (8 images), floor (4 images), roof (2 images), and wall (6 images) (Figure 2). The original color images and their height maps (both are 782 × 782 pixels) were reproduced from 3D textures (copyright free web site) [12]. The height maps undergone processing such as Gaussian filtering with a standard deviation of four and intensity adjustment. The reasons for this processing were to reduce black edges and reach the intended maximum elevation.

Based on the results of Kadyrova et al. [1], 2.5D prints of wood images were found to be perceived as more natural at 0.4 mm than at 0.6 mm and 0.8 mm. It is interesting to check whether 0.4 mm or elevation level around this number (i.e., 0.5 mm or 0.3 mm) provides a natural look to 2.5D prints of wood images. As a result, we varied elevation levels between 0 mm and 0.5 mm, meaning that each image had 6 reproductions. The reason for including flat prints (i.e., 0 mm) was to check if observers still prefer flat prints over elevated ones (i.e., 2.5D prints) or whether flat prints should be eliminated from the focus. The prints were fabricated with the OCE Arizona 2280GT 2.5D printer on an outdoor paper substrate. We used Alto printer mode (i.e., opaque elevation) and made print size 6.62 × 6.62 cm with an additional 0.3 cm on each side of the substrate paper for observers to hold the prints without touching their surface.

The visual experiment was conducted at two locations, UK and Norway, in English. We followed the same experiment design we used in our previous work [1]—first, we acquired consent from the observers followed by adaptation to illumination while the observers were reading instruction; afterwards, a training session was performed. A 3D-printed 45${}^{{}^{\circ}}$ holder was used to place the prints which were given in random order inside the light booth cabinet (Verivide CAC 60-5, illuminations were 1400 lux (Norway) and 1364 lux (UK)) with D65 illumination. The ranking experiment was conducted with instructions similar to those of our previous work [1]. We asked the observers to rank the 2.5D prints from the most to the least realistic representation of wooden wicker/floor/roof/wall and explain why. Thus, we gave keywords as a reference, and the observers were allowed to move and tilt the prints with provided gloves but not touch the surface. The distance between the eyes of the observers and the prints was approximately 50 cm. The observers were informed that there was no time restriction. The average duration of the experiment was 38 min per observer excluding the training session time (which was approximately 3 min per observer on average). With the exception of one observer, all other observers finished the experiment in one session. The audio responses of the observers were recorded for analysis purposes.

We had 21 observers (15 female and 6 male with an average age around 35 years and a standard deviation around 11 years) with normal color vision. We checked their color vision and visual acuity with Ishihara plates and a Snellen chart, respectively. There were 10 Asians and 11 Europeans who represented both naive and experienced (from computer science background) observers.

## 4. Results and Discussion

To determine what elevation makes 2.5D prints perceptually natural, we provide Z-scores (acquired from raw ranked data) of observers on the naturalness perception of the 2.5D prints at various elevation levels. We use an error bar plot to visualize Z-scores. Mean Z-scores are shown by a circle in the centre of the vertical lines. The Confidence Interval (*CI*) was derived using Equation (1) [13].

(1) $$CI=1.96\cdot \frac{\sigma }{\sqrt{N}},$$

where *N* is the number of observations, and σ is the standard deviation which in the case of Z-score can be computed as $1/\sqrt{2}$ [14]. 95% *CI* is the mean Z-scores ± *CI*. If two *CI* do not overlap, then there is a statistically significant difference between reproductions with 95% confidence.

**Figure 3:**
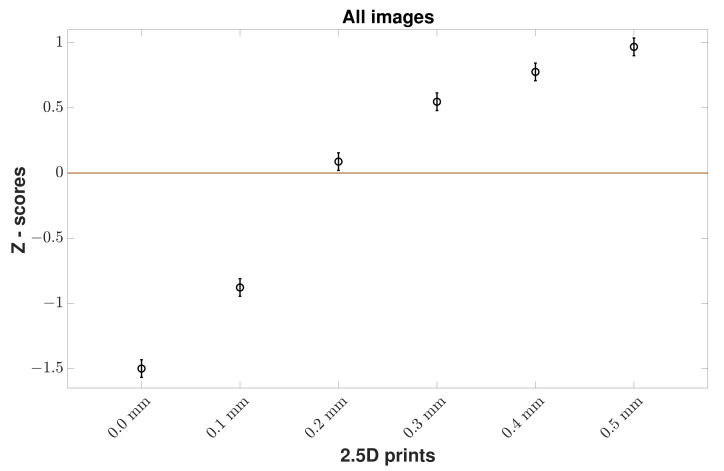
Z-scores of all wood images by all observers. Mean Z-score values for 2.5D prints at various elevation levels (x-axis) are given with 95% *CI*s. It shows that the observers found 2.5D prints with 0.5 mm as more natural looking than with no elevation.

From Figure 3 for the results for all wood images, we can see a clear trend that observers found higher elevation prints more natural than flatter ones. A binomial sign test was used on the raw data with Bonferroni correction (with a significance level of α/n, where α=0.05 is the desired alpha value and *n* is the number of comparisons: 0.05/15) [15] to check statistically significant differences between elevation levels. According to the *p*-values (Table 1), each elevation level had a statistically significant difference between each other. Based on the results, the optimal elevation that makes 2.5D prints of wood images look perceptually natural was 0.5 mm. However, if we consider the Z-scores of all the images in each wood content, then there was a slight variation regarding the optimal elevation in the case of floor and wall images while wicker and roof images showed that 0.5 mm is the optimal elevation. However, the overall trend that higher elevation prints are more natural than flatter ones stays the same whether we consider all wood images or images in each wood content. We present the results of each wood content image below.

**Figure 4:**
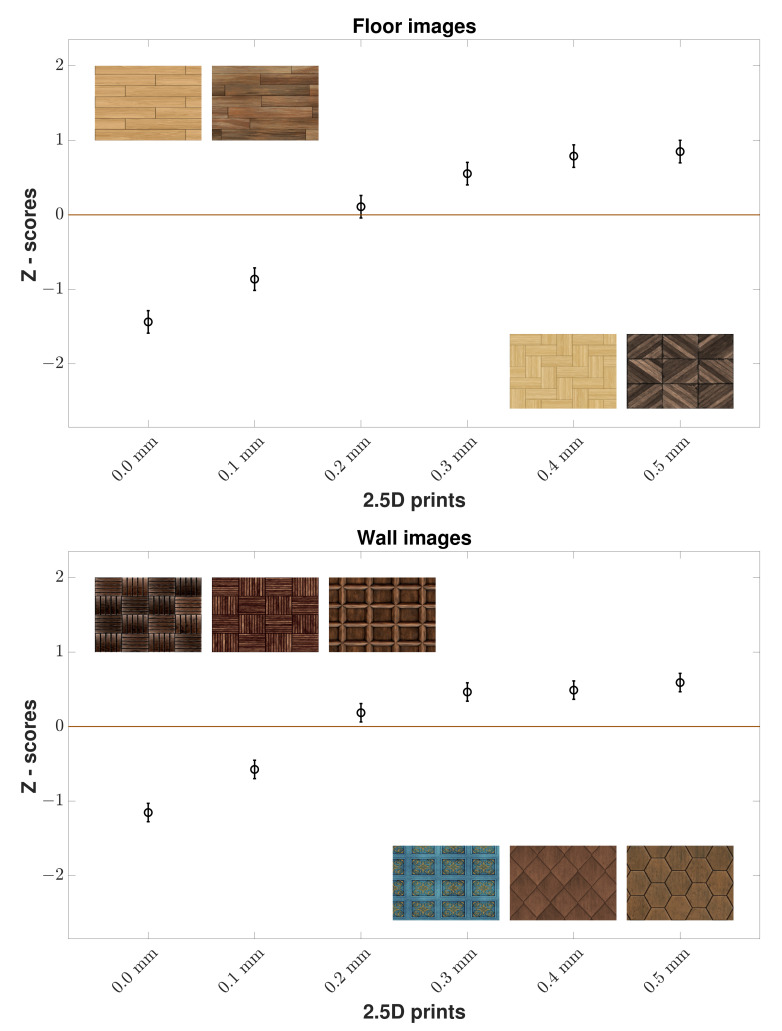
Z-scores of all wooden floor and all wooden wall images by all observers. Mean Z-score values for 2.5D prints at various elevation levels (x-axis) are given with 95% *CI*s. We can observe that for both floor and wall images, the observers found flat prints as the least natural. There are 4 floor and 6 wall images.

In the case of the *p*-values of all wooden floor images (Table 2), each elevation level had a statistically significant difference between each other except 0.4 mm and 0.5 mm. From Figure 4 for the results for all wooden floor images, we can see that 2.5D prints with higher elevation were found to be more natural than those at 0 mm by the observers.

In the case of the *p*-values of all wooden wall images, there was no statistically significant difference in elevation levels between 0.3 mm and 0.5 mm. From Figure 4 for the results for all wooden wall images, we can see that observers preferred 2.5D prints with higher elevation as more natural than those at 0 mm.

**Figure 5:**
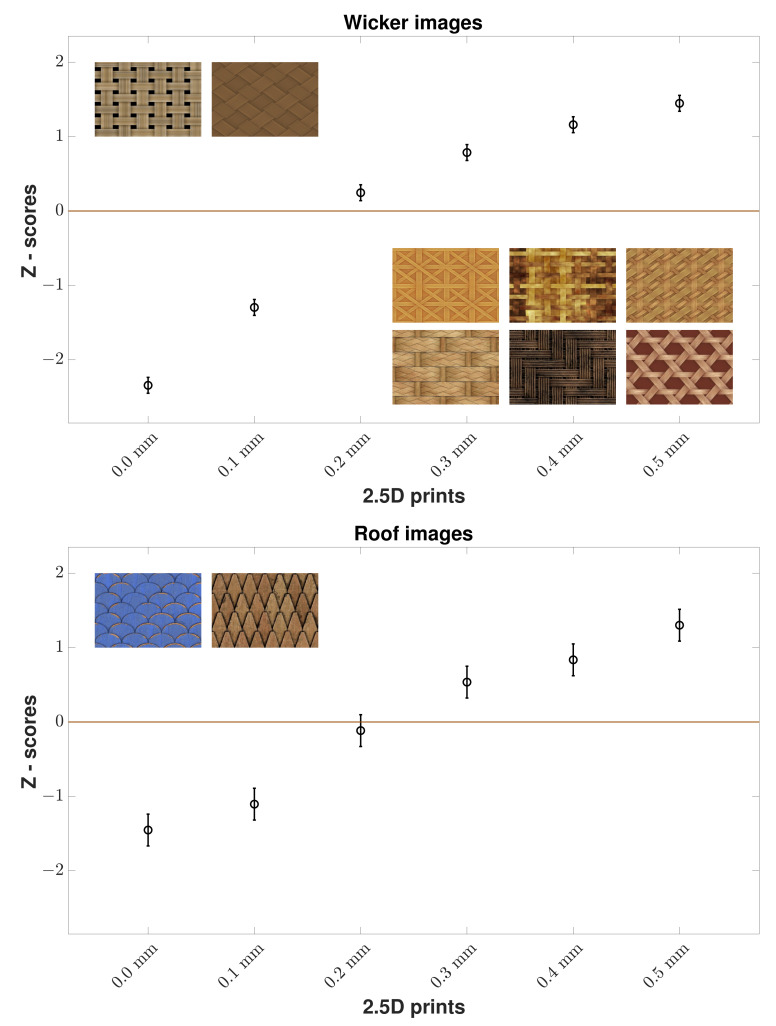
Z-scores of all wooden wicker and all wooden roof images by all observers. Mean Z-score values for 2.5D prints at various elevation levels (x-axis) are given with 95% *CI*s. We can observe that for both wicker and roof images, the observers found 2.5D prints with 0.5 mm as the most natural. There are 8 wicker and 2 roof images.

In the case of the *p*-values of all wooden wicker images, each elevation level had a statistically significant difference between each other. From Figure 5 for the results for all wooden wicker images, we can see that 2.5D prints with 0.5 mm were found to be more natural than those at 0 mm by the observers.

In the case of the *p*-values of all wooden roof images, each elevation level had a statistically significant difference between each other except 0.3 mm and 0.4 mm. From Figure 5 for the results for all wooden roof images, we can see that 2.5D prints with higher elevation were found to be more natural than those at 0 mm by observers. It is important to mention that we had a low number of images in wooden roof content.

As a result, we can observe that the optimal elevation that makes the 2.5D prints of wood images look perceptually natural is somewhat content-dependent. More specifically, when a certain elevation is reached for some content such as images of wooden floor or wall, that elevation is perceived as natural by the observers. In the case of images of wooden wicker and roof, higher elevation makes the most natural looking 2.5D prints.

Furthermore, we analyzed each observer’s Z-score in the case of all wood images and found that the majority preferred 0.5 mm as the one that makes the most natural looking 2.5D prints. We also checked inter-observer variability by Spearman correlation coefficient. On average for all images, majority of observers showed an agreement between each other on their rankings. This is different from the inter-observer variability results in Kadyrova et al.’s [1] work. We find this difference reasonable due to the following reasons: first, we used in our work only wood images while they used images of four material categories (wood, stone, glass, and metal); second, we varied only elevation while they varied elevation and surface roughness in the prints; last, the task in our case was somewhat easy for the observers to perceive differences between elevation levels than in their work. Additionally, we found similar performance when comparing the results for UK and Norway observers as well as between Asians and Europeans. The recorded audio data showed that most observers were able to find that the varying parameter was the elevation. They used a wide range of attributes and words to describe the elevation such as elevation, height, relief, depth, coming out, etc.

The limitation of our work is that it is based on images of wood only. Nevertheless, we provide a workflow that can be followed for other types of application where the output results may vary from application to application. In other words, the optimal elevation that makes 2.5D prints, for example, of stone images perceptually natural, might be different from the one found for wood images.

To conclude, it was clear that the flat prints do not look perceptually natural to observers. There should be elevation to make the prints look perceptually natural. In addition, we found that the content plays a role in finding the optimal elevation for the specific content of 2.5D prints of wood images to look perceptually natural. Moreover, there was a high correlation between majority of observers on their rankings which shows that the observers are rather consistent when assessing naturalness of 2.5D prints of wood images.

## 5. Conclusions and Future Work

The naturalness of 2.5D prints is affected by the level of elevation and is application-dependent. Therefore, we studied what elevation makes 2.5D prints of wood images to be perceived as natural. Various wood content images were considered such as wooden floor, wall, roof, and wicker. Based on the results, the optimal elevation is found to be content-dependent and in a range between 0.3 mm and 0.5 mm. More specifically, one can find the optimal elevation to use when certain elevation is reached for certain content of wood images. However, if one is interested in wood images regardless of content within wood images, then we found that 0.5 mm is the optimal elevation to use. Moreover, it was clear that observers found flat prints to be the least natural. Majority of observers showed a high correlation on their rankings, meaning that they are fairly consistent when it comes to 2.5D prints’ naturalness perception of wood images. Future work will be to study the naturalness perception of 2.5D prints further in terms of the effect of various types of ink because we hypothesize that the core effect on the naturalness perception of 2.5D prints might come from the ink itself that is used to fabricate the prints.

## Figures and Tables

**Table 1:** *p*-values obtained by a sign test for all wood images. Green cells are those that have a statistically significant difference. The threshold used in the Bonferroni correction is 0.05/15 = 0.0033.

	0.0 mm	0.1 mm	0.2 mm	0.3 mm	0.4 mm	0.5 mm
**0.0 mm**	-	$1.1602\times 10^{-52}$	$6.1431\times 10^{-78}$	$5.1105\times 10^{-74}$	$1.0257\times 10^{-71}$	$3.3530\times 10^{-70}$
**0.1 mm**		-	$1.8900\times 10^{-69}$	$5.8364\times 10^{-68}$	$7.0823\times 10^{-63}$	$2.2802\times 10^{-59}$
**0.2 mm**			-	$4.7035\times 10^{-41}$	$1.0702\times 10^{-37}$	$2.3412\times 10^{-39}$
**0.3 mm**				-	$1.7632\times 10^{-19}$	$2.0207\times 10^{-24}$
**0.4 mm**					-	$8.2898\times 10^{-14}$
**0.5 mm**						-

**Table 2:** *p*-values obtained by a sign test for all wooden floor images. Green cells are those that have a statistically significant difference whereas red cells are those that do not have a statistically significant difference. The threshold used in the Bonferroni correction is 0.05/15 = 0.0033.

	0.0 mm	0.1 mm	0.2 mm	0.3 mm	0.4 mm	0.5 mm
**0.0 mm**	-	$7.8639\times 10^{-11}$	$2.0973\times 10^{-19}$	$3.4018\times 10^{-18}$	$3.4018\times 10^{-18}$	$5.1376\times 10^{-16}$
**0.1 mm**		-	$4.5431\times 10^{-17}$	$5.1376\times 10^{-16}$	$4.3086\times 10^{-14}$	$4.3086\times 10^{-14}$
**0.2 mm**			-	$1.9382\times 10^{-9}$	$1.3329\times 10^{-7}$	$8.5423\times 10^{-9}$
**0.3 mm**				-	$2.6645\times 10^{-4}$	$1.0715\times 10^{-4}$
**0.4 mm**					-	0.2299
**0.5 mm**						-

## Data Availability

The original images and their height maps used in this work can be found at https://3dtextures.me, accessed on 6 July 2021.
